# Social Frailty in Older People: A Concept Analysis

**DOI:** 10.15649/cuidarte.4939

**Published:** 2025-09-26

**Authors:** Javier Rojas-Avila, Alejandra Ximena Araya, Nicole Pinilla Carrasco

**Affiliations:** 1 Faculty of Health Sciences, Universidad Autónoma de Chile. Cinco Poniente 1670, Talca, Chile. E-mail: javier.rojas@uautonoma.cl Universidad Autónoma de Chile Talca Chile javier.rojas@uautonoma.cl; 2 Faculty of Nursing, Universidad Andrés Bello, Santiago, Chile. Associate Researcher, Millennium Institute for Research on Care (MICARE). Principal Investigator, ANID-FONDECYT [1241671], Santiago, Chile. E-mail: alejandra.araya.g@unab.cl Universidad Andrés Bello Santiago Chile alejandra.araya.g@unab.cl; 3 Faculty of Medicine, Department of Nursing Science, Universidad Católica de la Santísima Concepción, Concepción, Chile. Doctoral Program in Nursing Science, Faculty of Nursing, Universidad Andrés Bello, Santiago, Chile. E-mail: ni.pinilla@uandresbello.edu Universidad Católica de la Santísima Concepción Santiago Chile ni.pinilla@uandresbello.edu

**Keywords:** Frailty, Aged, Systematic Review, Health of the Elderly, Geriatrics, Fragilidad, Persona Mayor, Revisión Sistemática, Salud de la Persona Mayor, Gerontología, Fragilidade, Idoso, Revisão Sistemática, Saúde do Idoso, Geriatria

## Abstract

**Introduction:**

Social frailty is a multidimensional concept that includes general and social resources, activities, and the fulfillment of needs; understanding and assessing it helps guide interventions for older adults.

**Objective:**

To clarify the concept of social frailty in older adults to improve its application in research, clinical practice, and education.

**Materials and Methods:**

The Walker and Avant method was used to analyze social frailty in older adults, following stages such as concept selection, purpose definition, identification of uses, attributes, and related cases. Systematic searches were conducted in PubMed, ScienceDirect, Web of Science, and CINAHL, along with manual searches of references and dictionaries.

**Results:**

Social frailty in older adults is primarily defined as the loss of social resources, reduced ability to meet basic social needs, and diminished participation in meaningful activities such as maintaining close relationships, engaging in volunteering, having an occupation (paid or unpaid), and participating in religious or community activities. Factors such as loneliness, isolation, and lack of social support are associated with negative health outcomes.

**Discussion:**

Social frailty must be addressed from an interdisciplinary perspective. Health professionals should play an active role in identifying social frailty and implementing community-based strategies, psychosocial programs, and support networks that strengthen inclusion and social connection, thereby contributing to the prevention of functional and emotional decline.

**Conclusion:**

Social frailty impacts the physical, mental, and emotional health of older adults, underscoring the importance of inclusive, preventive, and care-centered policies.

## Resumen

**Introducción:**

La fragilidad social es un concepto multidimensional que incluye recursos generales y sociales, actividades y satisfacción de necesidades; comprenderla y evaluarla permite orientar intervenciones en personas mayores.

**Objetivo:**

Clarificar el concepto de fragilidad social en personas mayores para mejorar su aplicación en investigación, práctica clínica y educación.

**Materiales y métodos:**

Se empleó el método de Walker y Avant para analizar la fragilidad social en personas mayores, siguiendo etapas como la selección del concepto, definición del propósito, identificación de usos, atributos y casos relacionados. Se realizaron búsquedas sistemáticas en PubMed, ScienceDirect, Web of Science y CINAHL, así como búsquedas manuales en referencias y diccionarios.

**Resultados:**

La fragilidad social en personas mayores se define principalmente como la pérdida de recursos sociales, habilidades para satisfacer necesidades sociales básicas y participación en actividades significativas, como mantener relaciones cercanas, realizar voluntariado, tener una ocupación (remunerada o no), participar en actividades religiosas o comunitarias. Factores como la soledad, el aislamiento y la falta de apoyo social se asocian con efectos negativos en la salud.

**Discusión:**

La fragilidad social requiere ser abordada desde una perspectiva interdisciplinaria. Los profesionales de la salud deben desempeñar un rol activo en su identificación y en la implementación de estrategias comunitarias, programas psicosociales y redes de apoyo que fortalezcan la inclusión y el vínculo social, contribuyendo a la prevención del deterioro funcional y emocional.

**Conclusiones:**

La fragilidad social afecta la salud física, mental y emocional de personas mayores, resaltando la necesidad de políticas públicas inclusivas, preventivas y centradas en el cuidado.

## Resumo

**Introdução:**

A fragilidade social é um conceito multidimensional que inclui recursos gerais e sociais, atividades e satisfação de necessidades. Compreendê-la e avaliá-la auxilia na orientação de intervenções para idosos.

**Objetivo:**

Esclarecer o conceito de fragilidade social em idosos para aprimorar sua aplicação em pesquisa, prática clínica e educação.

**Materiais e métodos:**

O método de Walker e Avant foi utilizado para analisar a fragilidade social em idosos, seguindo etapas como seleção de conceitos, definição de propósito, identificação de usos, atributos e casos relacionados. Buscas sistemáticas foram realizadas no PubMed, ScienceDirect, Web of Science e CINAHL, bem como buscas manuais em referências e dicionários.

**Resultados:**

A fragilidade social em idosos é definida principalmente como a perda de recursos sociais, da capacidade de atender às necessidades sociais básicas e da participação em atividades significativas, como manter relacionamentos próximos, ser voluntário, ter uma ocupação (remunerada ou não) e participar de atividades religiosas ou comunitárias. Fatores como solidão, isolamento e falta de apoio social estão associados a desfechos negativos em saúde.

**Discussão:**

A fragilidade social requer uma abordagem interdisciplinar. Os profissionais de saúde devem desempenhar um papel ativo na sua identificação e implementação de estratégias comunitárias, programas psicossociais e redes de apoio que fortaleçam a inclusão e os laços sociais, contribuindo para a prevenção do declínio funcional e emocional.

**Conclusões:**

A fragilidade social afeta a saúde física, mental e emocional dos idosos, evidenciando a necessidade de políticas públicas inclusivas, preventivas e centradas no cuidado.

## Introduction

Frailty is a concept that has gained attention in the scientific literature, particularly in the context of global population aging. It is defined as a state of heightened vulnerability to internal and external stressors, which increases the risk of negative health consequences^1^,^2^. Although social frailty is preventable and treatable^3^,^4^, the theoretical understanding of the concept and its empirical assessment using validated instruments remain contested issues, reinforcing its importance in the field of public health^1^,^5^.

The study of frailty has advanced considerably, highlighting the need for a clear conceptual framework^6^. There are various assessment tools that vary in terms of criteria and contexts of application^7^-^10^. Conceptual models such as the frailty phenotype^11^ and the accumulation of deficits model^12^ have been fundamental in developing more comprehensive approaches that address the different dimensions of this phenomenon^13^.

Social frailty, one of the least researched and most complex subdomains within the multidimensional construct of frailty in older adults^14^, has been associated with various adverse health conditions, such as disability, cognitive impairment, and depressive symptoms^15^,^16^. These associations highlight the need for a deeper understanding of its components and mechanisms, especially in the context of demographic aging. Accordingly, this analysis aims to clarify the concept of social frailty in community- dwelling older adults, with the goal of facilitating its application in research, clinical practice, and the design of preventive interventions^17^.

The ambiguity in the definition and operationalization of social frailty, reflected in the diversity of criteria such as isolation, loneliness, social networks and support, and participation, highlights the need for conceptual clarification to improve understanding and guide effective interventions^18^. Social frailty has since been approached from multiple perspectives. Previous research has highlighted different approaches, including the individual's social functioning and connections^15^, the decline of relationships and social support^19^, as well as social roles, networks, and activities^20^.

The variability across definitions highlights the dynamic and context-dependent nature of social frailty, influenced by socioeconomic and cultural factors. A clear and shared understanding of the concept is essential for developing effective policies and strategies to address social vulnerabilities among older adults. Clarifying the concept enables more accurate analysis of its causes and consequences, and provides the foundation for strengthening support networks and promoting social well-being. From a research perspective, this analysis contributes to establishing a more robust theoretical framework to guide future empirical research, especially by identifying the key dimensions of the phenomenon and formulating criteria that facilitate its application in different contexts. This endeavor is a priority for scholars, policymakers, and practitioners in the social and health fields.

Walker and Avant proposed that concept clarification is achieved by examining its uses, attributes, antecedents, consequences, and empirical referents, a common approach to defining concepts^21^. This study focused on clarifying the concept of social frailty in community-dwelling older adults, who are particularly exposed to social conditions that hinder community integration and reduce participation in daily life. Although this form of frailty can be observed in other groups, such as individuals with disabilities, it has specific implications for older people, given its association with increased risks of dependency, functional decline, and social exclusion. These characteristics justify the need for a targeted conceptual analysis to inform research, the design of interventions, and training in gerontology.

## Materials and Methods

**Concept analysis method**

The Walker and Avant method was applied to analyze the concept of social frailty in older adults^21^. This systematic and reliable approach is widely used in nursing research. The analysis, conducted in eight steps, involved identifying antecedents, attributes, consequences, and empirical referents, as well as constructing cases. The steps included: selecting the concept; establishing the purpose; identifying uses; defining attributes; recognizing model, borderline, and contrary cases; and determining antecedents, consequences, and empirical referents.

**Selecting the concept**

The selection of the concept of social frailty in community-dwelling older adults is fundamental, as it provides a solid conceptual basis for advancing its study and application in the contexts of public health and aging. A clear definition enables the identification of its essential components and an understanding of its implications for the well-being of this population group. From a research perspective, concept analysis contributes to a more accurate and consistent understanding, which facilitates the development of coherent, comparable, and better-grounded studies. At the theoretical level, it contributes to consolidating a still-evolving construct by integrating social dimensions often underestimated in traditional frailty models. Finally, in clinical and community practice, a precise understanding of the concept enables more accurate identification of situations of social vulnerability in older adults and informs interventions that promote inclusion, autonomy, and quality of life.

**Determining the purpose of the concept analysis**

The purpose of this analysis is to clarify the concept of social frailty in community-dwelling older adults in order to enhance its understanding and guide its application in research, clinical practice, and health education. Although social frailty has also been mentioned in other groups, such as people with disabilities or chronic illnesses, it is particularly relevant in older adults due to its association with social isolation, loss of roles, decline in accumulated social capital, and increased dependency. Clarifying this concept in the context of aging contributes to theoretical development, identification of its key dimensions, and generation of evidence to support the future development of assessment and intervention strategies that are more relevant to this population group.

**Identification of the uses of the concept**

**Data sources**

Systematic searches were conducted in PubMed, ScienceDirect, Web of Science, and CINAHL using the term “social frailty” from January 1, 2010, to September 30, 2024. This period was chosen to cover the most relevant publications of recent years, considering the growing presence of the concept in the scientific literature. In addition, a dictionary was consulted, and manual searches were conducted in the reference lists of relevant studies.

**Dictionaries**

The Diccionario de la Lengua Española of the Real Academia Española (DRAE) was consulted online, as it is the authoritative source for the Spanish language and is widely used in health research. Specialized dictionaries, such as those in philosophy, were excluded, as the analysis aimed to clarify the use of the concept of “social frailty” in applied contexts, particularly in public health and gerontology, rather than from a philosophical or epistemological perspective.

**Eligibility criteria**

Publications in English, Spanish, and Portuguese that provided definitions, attributes, antecedents, or consequences of social frailty were included, as well as qualitative, quantitative, or systematic reviews explicitly mentioning the term “social frailty.” Articles addressing assessment in community-dwelling older adults were also considered. Duplicates, editorials, conference abstracts, inaccessible texts, literature on hospitalization of older adults, and scoping reviews were excluded.

**Data collection and analysis**

A systematic literature search was conducted following standard review procedures. Two authors independently selected studies in a two-stage process, resolving disagreements through discussion and consultation with a third reviewer. The data extracted from the studies supporting the following concept analysis are available for free access and consultation in Mendeley Data^22^.

**Figure 1:**
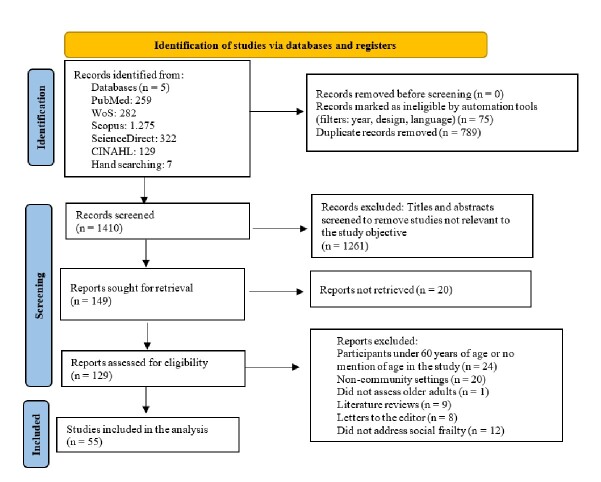
PRISMA article selection flow diagram

*Note: Developed by the authors based on Page et al.^23^*

## Results

As part of the methodological process proposed by Walker and Avant for concept analysis^21^, a systematic literature search was conducted to identify the different uses and applications of the term “social frailty.” A total of 2,267 studies were found in online databases, and 7 through hand searching. After removing duplicates, 1,410 titles and abstracts were screened, of which 1,261 were excluded. Full texts for 149 records were sought for retrieval, of which 55 studies were finally selected for providing relevant information for the stages of analysis (attributes, antecedents, consequences, and empirical referents). These studies served as the basis for a rigorous and structured conceptual analysis. 

**Dictionary definition**

Walker and Avant pointed out that identifying all possible uses of the concept is the first step in analyzing its attributes^21^. Although the term “social frailty” does not appear in the Diccionario de la Lengua Española of the Royal Spanish Academy, the related terms “social” and “frailty” were identified. “Social” is defined as “pertaining or relating to a company or society, or to partners, colleagues, allies, or confederates,” and “frailty” as “the quality of being frail” ^24^,^25^. 

The most prevalent definition of social frailty is that proposed by Bunt et al.^14^, who describe it as a multidimensional concept involving a continuum of being at risk of losing, or having lost social resources, activities, or abilities essential for fulfilling basic social needs. This approach is based on the theory of social production functions, which identifies three fundamental needs: affection, behavioral confirmation, and status^26^. 

The definition of social frailty proposed by Bunt et al.^14^ considers four key factors: social resources (e.g., support networks), social needs (e.g., social support), social behaviors or activities (e.g., social participation), and general resources (e.g., educational level and financial status). This conceptualization has been supported by a systematic review by Hamid et al.^27^, which confirmed the relevance of these categories in the assessment of social frailty. 

Regarding the operational definition of social frailty, Makizako et al.^15^ proposed an approach based on five elements: going out less frequently, visiting friends sometimes, feeling helpful to friends or family, living alone, and talking with someone every day. The applicable responses are classified as: no social frailty (0 responses), prefrailty (1 response), and social frailty (≥ 2 responses). This approach is one of the most widely used in the literature on social frailty. 

Although interest in social frailty has grown, significant conceptual gaps persist. There is no consensus on whether it constitutes an autonomous domain, its precise place in frailty models, or the criteria that best define it. Furthermore, it is often conflated with concepts such as social vulnerability, and current definitions combine structural dimensions and individual perceptions without a unified framework. In older adults, where aging interacts with various social determinants, this lack of clarity limits its clinical and community applications. Therefore, a rigorous concept analysis is required to clarify its definition and strengthen its use in research, professional practice, and intervention design. 

**Table 1:** Summary of the different uses of the concept of social frailty

Sources	Type of source	Definition	Attributes	Antecedents	Consequences	Population	Empirical referent
Makizako et al., 2015^15^	Quantitative study	(-)	The attributes of social frailty, according to the questionnaire used, include a decrease in social activity, a decline in the quality of interpersonal relationships, and a change in the perception of social role. Specifically, it assesses whether individuals have reduced the frequency of their outings, the number of visits to friends, and whether they feel useful to others. Factors such as boredom, living alone, and the frequency of daily interactions, both by phone and in person, are also considered. Negative responses to these questions indicate a higher level of social frailty, reflecting the impact on the social and emotional life of the older person.	(-)	Increased risk of disability.	Individuals aged ≥65 years	7-item questionnaire
Park et al., 2019^16^	Quantitative study	Bunt et al. recently proposed that social frailty is a continuum of being at risk of losing, or having lost general or social resources, social behaviors and activities, and self-management abilities.	(-)	(-)	Increased risk of disability	Individuals aged ≥65 years	Makizako's 5-item questionnaire
Makizako et al., 2018^17^	Quantitative study	(-)	Domains of social frailty, including social roles, networks, and social activities in older adults, may require higher levels of functioning.	(-)	Cognitive decline, dementia. Physical frailty.	Individuals aged ≥65 years	5-item questionnaire
Bunt et al., 2021^26^	Qualitative study	Social frailty can best be conceptualized as a multidimensional concept referring to a variety of general and/or social resources (or restrictions), social behaviors and activities, and self-management abilities, which all have a function in adding to (or affecting) social needs fulfillment.	(-)	(-)	(-)	Individuals aged ≥65 years	(-)
Hamid et al., 2024^27^	Systematic review	Social frailty can be defined as the loss of one or more human social resources essential for fulfilling basic human needs throughout life.	A lack of frequent participation in social events, networks, and contact, and insufficient support, leading to serious health outcomes. Social needs, social resources (such as social supports and social networks), social behaviors or social activities, and general resources (indirect way of fulfilling social needs, such as living situation, educational level, and income or financial status).	Reduced participation in social and community activities, such as volunteering, visiting family and friends, attending social events, and participating in social community clubs, can lead to social frailty	Mortality, physical frailty, and disability incidence	Individuals aged ≥60 years	16 different instruments
Huang et al., 2020^28^	Quantitative study	Social frailty based on Blunt’s theoretical framework, which considers aspects such as general resources, social resources, social behavior, and the fulfillment of basic social needs	(-)	(-)	(-)	Individuals aged ≥60 years	4-item questionnaire
Yamada et al., 2018^29^	Quantitative study	It includes general resources, social resources, social behavior, and fulﬁllment of basic social needs.	(-)	(-)	(-)	Individuals aged ≥65 years	4-item questionnaire
Tsutsumimoto et al., 2018^30^	Quantitative study	Going out less frequently compared with last year, not visiting friends sometimes, not feeling helpful to friends or family, living alone, and not talking with someone every day.	(-)	(-)	Development of depressive symptoms	Individuals aged ≥60 years	5-item questionnaire
Doi et al., 2022^31^	Quantitative study	The definition considered five items: (1) less frequent occurrences of going out compared to the preceding year (applicable response: yes), (2) visiting friends occasionally (applicable response: no), (3) considering oneself helpful to friends or family (applicable response: no), (4) living alone (applicable response: yes), and (5) talking with someone every day (applicable response: no).	(-)	(-)	(-)	Individuals aged ≥60 years	5-item questionnaire
Ma et al., 2018^32^	Quantitative study	It can be defined as the absence of social resources, social activities, and self-management abilities that are important for fulfilling basic social needs.	Unhelpful to others, limited social participation, loneliness, financial difficulty, and not having anyone to talk to.	Participants were widowed, living alone, and current smokers; they used alcohol and did not drink tea. Used to be engaged in heavy physical labor as their occupation, and were not currently engaged in any work. Participants physically frail, with dementia, who had experienced a recent life event, subjective memory decline, depression, and cognitive decline.	Predicts mortality	Individuals aged ≥60 years	5-item scale
Bae et al., 2018^33^	Quantitative study	The operational deﬁnition of social frailty involves assessing the risk of disability due to lower social engagement status in older adults.	Going out less frequently compared with last year (yes), visiting friends sometimes (no), feeling helpful to friends or family (no), living alone (yes), and talking to someone every day (no).	Hearing problems	(-)	Individuals aged ≥65 years	5-item scale
Zare et al., 2024^34^	Quantitative study	Social frailty refers to a lack of social resources, social activities, and self-management abilities to meet people’s social needs.	They include social activity, which is reflected in how often a person goes out and interacts with others, as well as the ability to maintain interpersonal relationships, assessed through interaction with friends and daily communication. Social support, measured by the willingness to turn to family or friends for advice, and daily interaction, which involves sharing at least one meal a day with someone, are also considered. In addition, confidence in having someone to lean on is crucial, along with access to resources, which manifests itself in limitations on the use of financial resources for medical services.	(-)	(-)	Individuals aged ≥60 years	8-item Social Frailty Scale.
Li et al., 2024^35^	Quantitative study (meta-analysis)	Social frailty is a concept that refers to the lack or loss of social resources, behaviors, activities, and self-management skills needed to fulfill fundamental social needs	These social needs include emotional needs, the need for behavior to be recognized, and the need for identity roles to be recognized.	Depression, activities of daily living, physical inactivity, motor deficits, cognitive impairment, and physical frailty	Physical decline, mood disorders, and predictive of death	Individuals aged ≥60 years	11 different instruments
Zhang et al., 2024^36^	Quantitative study	It is defined as a continuum of being at risk of losing or having lost resources that are important for fulfilling one or more basic social needs during the lifespan.	Social frailty is an integrated construct including multiple social aspects, such as social behaviors (e.g., volunteer work, participating in club or group activities), social resources (e.g., living with spouses or partners), and basic social needs (e.g., financial status and social support).	(-)	Motoric cognitive risk syndrome (MCR), disability, Alzheimer's disease, depression, and mortality.	Individuals aged ≥60 years	Makizako's 5-item questionnaire
Dong et al., 2024^37^	Quantitative study	Social frailty is described as a state of deficiency in critical general and social resources, social behaviors, as well as self-management abilities essential for satisfying one’s social needs.	Social resources, social activities, and financial resources, and the fulfillment of social needs.	Avoid social activities, participate less in social activities, and shrink their social circle.	(-)	Individuals aged ≥60 years	8-item Social Frailty Scale.
Shah et al., 2023^38^	Quantitative study	A lack of resources to fulfill older adults’ basic social needs.	General resources, social resources, social activities, and fulfillment of basic social needs.	(-)	(-)	Individuals aged ≥60 years	Social Frailty Index
Damasceno et al., 2023^39^	Quantitative study	A continuous risk and/or loss of resources that are important for the fulfillment of one or more basic social needs during older adults’ lives.	It includes domains of economic status, social networks, and social activities. Inability to help others, limited social participation, loneliness, financial difficulty, and not having anyone to talk to.	(-)	Predictor of mortality	Individuals aged ≥65 years	HALFT scale
Qi et al., 2023^40^	Quantitative study	It is conceptualized as being at risk of losing or having lost sufficient social support, activities, or resources required to fulfill basic social needs.	Social activities, social support, social networks, loneliness, and living alone. Inability to help others, limited social participation, loneliness, and financial difficulty.	(-)	Disability, mortality	Individuals aged ≥60 years	HALFT scale
Sun et al., 2023^41^	Quantitative study	(-)	General and social resources, social behaviors, and the satisfaction of basic social requirements. (1) financial support: “Is your annual per capita income of households <RMB10,000?” (yes = 1 point, no = 0 points), (2) living status: “How many people do you live with?” (0 = 1 point, ≥1 = 0 points), (3) social activity: “Do you participate in any community activities regularly?” (no = 1 point, yes = 0 points), and (4) social contact: “Do you sometimes visit your friends and relatives?” (no = 1 point, yes = 0 points).	Advanced age	(-)	Individuals aged ≥65 years	Social Frailty Index
Uchida et al., 2023^42^	Quantitative study	A condition in which the multiple social domains are weakened, including a variety of general and social resources, social networks, social behaviors, activities, and self-management abilities.	(-)	(-)	(-)	Individuals aged ≥65 years	Makizako's 5-item questionnaire
Choi et al., 2022^43^	Quantitative study	Social frailty is defined as a continuum of being at risk of losing, or having lost, resources that are important for fulfilling one or more basic social needs during older adults’ lifespan.	(1) going out (not participating in any leisure and social activities, such as travel, hobbies, learning or studying, social clubs, networking, political and social groups, volunteering, senior citizen centers, community centers for older adults); (2) visiting friends (no); (3) feeling worthless (yes); (4) living alone (yes); and (5) contact with someone (not communicating with relatives including siblings, friends, neighbors, and acquaintances by phone, text message, or e-mail).	(-)	(-)	Individuals aged ≥65 years	Makizako's 5-item questionnaire
Ko et al., 2021^44^	Quantitative study	Social frailty is defined as a continuum of being at risk of losing, or having lost, resources that are important for fulfilling one or more basic social needs during the lifespan.	Going out (not participating in any leisure and social activities, such as travel, hobbies, learning or studying, social clubs, networking, political and social groups, volunteering, senior citizen centers, community centers for older adults), visiting friends (no), feeling worthless (yes), living alone (yes), and contact with someone (no).	(-)	(-)	Individuals aged ≥65 years	Makizako's 5-item questionnaire
Ko et al., 2022^45^	Quantitative study	Social frailty among older adults reflects a spectrum of increased risk or having lost resources required to meet one or more basic social needs during their lifespan.	Makizako and colleagues defined social frailty using five items such as going out less frequently, rarely visiting friends, feeling unhelpful to friends or family, being alone, and not conversing with someone every day.	(-)	(-)	Individuals aged ≥60 years	Makizako's 5-item questionnaire
Kodama et al., 2022^46^	Quantitative study	In recent years, social frailty has been defined provisionally as the absence of social resources, social activities, and self-management abilities for fulfilling basic social needs.	Makizako and colleagues defined social frailty based on five attributes: (i) living alone, (ii) not talking with someone every day, (iii) not feeling helpful to friends or family, (iv) going out less frequently compared with last year, and (v) not visiting friends sometimes.	Depressive symptoms	(-)	Individuals aged ≥65 years	Makizako's 5-item questionnaire

** Related terms**

Related concepts are those terms that share similar characteristics or aspects with the main concept but are not identical to it. These concepts serve to broaden or better contextualize the analysis^21^.

**Social vulnerability**

Social frailty and social vulnerability are related concepts, but they differ in their scope. Social frailty refers to a condition in which a person has begun to lose, or is at risk of losing, the social resources, abilities, or activities necessary to meet their basic needs^14^. This loss compromises the individual's ability to cope with social stressors, placing them on a path of functional decline and isolation.

In contrast, social vulnerability describes a broader condition of accumulated susceptibility to adverse social factors, which can predispose individuals to negative health outcomes even before tangible losses of resources occur^47^-^49^. It is often assessed using indices that incorporate variables such as socioeconomic status, support network, access to services, and other structural conditions.

Thus, while social frailty implies a specific and observable deterioration in essential social components (i.e., an active manifestation of risk), social vulnerability reflects a latent or structural state of heightened susceptibility to experience harm during adverse situations^50^.

Both concepts are associated with higher mortality rates, cognitive decline, and functional dependency^51^,^52^. However, they differ in focus: frailty centers on the individual and their immediate environment, whereas vulnerability adopts a broader, cumulative perspective of social disadvantages that affect health throughout the life course.

**Social prefrailty**

Social frailty and social prefrailty are related but distinct concepts related to well-being of older adults. Social frailty is characterized by negative indicators, such as a lack of participation in social activities, loneliness, and limited interaction, with two or more indicators present, thereby adversely affecting quality of life and health^14^. In contrast, prefrailty is an earlier stage, with only one of these indicators observed, but there is a greater risk of progression to frailty without preventive measures^15^.

**Attributes**

Defining attributes are those key characteristics that distinguish a concept from others that are similar to it. They should be the most recurrent in instances of the concept and reflect its core meaning without including unnecessary characteristics^21^.

**Social resources**

Social resources are essential for meeting social needs, as they facilitate their fulfillment^14^. Social networks, as a key component, include interpersonal relationships, the bonds formed, and aspects such as network size, frequency of interaction, diversity, and reciprocity^53^. A strong family and social network can significantly improve life satisfaction in older adults^54^.

Research has shown a significant connection between social networks and frailty in older adults. A study reported that frail older adults have smaller social networks and higher levels of loneliness^55^. Another study found that an unsatisfactory social network increases the risk of physical frailty in women, though not in men^56^. In Japan, older adults with low social engagement were 52% more likely to experience severe frailty^57^, and the absence of close contacts was correlated with greater social frailty^58^. This evidence highlights the importance of quality social networks in preventing frailty in older age.

**Social behaviors and activities**

Social behaviors refer to actions directed toward satisfying social needs^14^. This study focuses on social participation, understood as the integration of older adults into institutions, associations, and community networks. Belonging to social groups is associated with a sense of belonging and social support^59^. Social participation serves as a community support resource, offering emotional, instrumental, and informational support, thereby buffering stress, and improving physical and mental health in older age^59^.

Social participation is key to preventing and managing frailty in older adults, thereby improving their well-being. Participation in activities such as paid work, volunteering, or neighborhood associations reduces the risk of frailty^60^. The frequency and type of participation are decisive factors, with regular engagement in activities and occasional volunteering being associated with lower risks^61^. The use of technology and playing sports are also linked to lower levels of frailty^62^. Social participation, both community and family-based, promotes involvement in social activities, and its absence is associated with greater social frailty^63^. Encouraging active and diverse participation is crucial to improving the quality of life and preventing frailty in older adults^41^,^64^.

**Social support**

Social support is understood as well-being derived from the availability and use of social and general resources, as well as from social activities that contribute to satisfaction^14^. It encompasses interactions of assistance, affection, or affirmation^65^ and is classified into three types: emotional, informational, and instrumental, coming from informal networks (e.g., family, friends) and formal networks (e.g., community groups)^66^,^67^.

Perceived social support is crucial for older adults, as it reduces isolation and encourages healthy behaviors^68^,^69^. It also provides a sense of purpose and offers feedback on health, thereby contributing to longevity. Social support networks are essential at all stages of life, especially in older age, which is marked by loss and the need to forge new bonds^70^. Various studies highlight the heterogeneity of these networks, emphasizing the importance of family and friendship relationships^71^,^72^.

A study in Medellín identified key factors for adequate social support, such as living with more than four family members and maintaining a network of close friends^73^. Similarly, a study conducted in Chile during the pandemic showed positive perceptions of social support, although friends' ability to provide support was lower^74^. These findings highlight the importance of social support for mental and emotional health^42^,^75^.

Social support also has a direct influence on frailty prevention and management. In China, higher levels of social support were found to reduce psychological distress and the risk of cognitive frailty^76^. In Berlin, the lack of social support increased the risk of frailty, although it was not directly related to the transition to frailty^77^. Perceived social support, particularly from close family members, can help mitigate frailty and improve mental health^78^. Other studies in China, Japan, and South Korea indicated that emotional and instrumental support reduces the risk of physical and social frailty^58^,^79^.

In summary, both perceived and received social support are essential for preventing and managing frailty in older adults. Evidence suggests that strengthening social networks, reducing isolation, and improving social support are key factors in improving health and well-being in older age.

**Identifying a model case**

Elena, 78, has been living alone in her home for three years, following the death of her husband, with whom she shared more than five decades of marriage. For much of her adult life, Elena assumed multiple social roles: she worked as an elementary school teacher, served as an active leader in her neighborhood association, and volunteered at her local parish. Her social life was rich and structured: she organized gatherings, attended community workshops, and maintained a steady network of friends and colleagues with whom she went out regularly. After becoming widowed, however, Elena’s social life began to decline progressively. Her support network has diminished, and she currently maintains regular contact only with her daughter, who visits twice a week, and occasional communication with a couple of friends. Elena has stopped participating in community activities, partly due to mobility issues and partly because of lack of motivation and interest. She has lost the habit of going out frequently, and interaction with neighbors is minimal. She no longer feels part of the community to which she once actively contributed, and reports feeling lonely and disconnected. Based on Walker and Avant's concept analysis method^21^, this case illustrates all the attributes of social frailty: loss of social resources, decline in meaningful social activities, and reduction in social support, as well as loss of the ability to sustain interactions that fulfill basic social needs. Additionally, it shows emotional and functional consequences associated with this condition.

**Identifying borderline, related, and contrary cases**

**Related case**

Carmen, 82, currently lives with her son, who attends to her daily needs and accompanies her in her everyday life. During her adult life, Carmen worked as a school secretary and was an active member of her community: she participated in a book club, regularly attended parish meetings, and maintained a wide network of friends with whom she shared outings and recreational activities. She also frequently cared for her grandchildren and organized family gatherings. Over time, and following knee surgery that significantly reduced her mobility, Carmen’s participation in social activities declined. Her network of contacts has also diminished, although she maintains regular communication with two close friends. Her son has tried to keep her connected to her social environment by arranging visits from family and friends, which she greatly values. Despite these limitations, Carmen preserves a strong emotional bond with her son, who provides her with continuous emotional and practical support. Although she expresses sadness about not being able to participate in her community as actively as she did before, her situation does not constitute social frailty, since she retains essential resources that enable her to meet her basic social needs. In this case, some attributes of social frailty are partially present; however, strong family support functions as a protective factor against further social decline.

**Borderline case**

Teresa, 73, has been living alone in an apartment for a year, after moving to a new city to be closer to healthcare services. For most of her life, she worked as a seamstress from home, taking orders from neighbors and actively participating in local fairs. She maintained a social life connected to her community: she spent time with regular clients, attended meetings at the mothers' center, and collaborated in charitable activities with other women in the area. In recent years, especially following the pandemic and her move, Teresa has been unable to establish new meaningful social connections in her new environment. Her current interactions are limited to sporadic phone calls with her brother, who lives in another city, and brief exchanges with a neighbor in her building. She does not participate in community activities or receive frequent visitors. Although she has internet access, she does not feel comfortable using social media or other digital platforms to socialize. This limited interaction has begun to affect their emotional well-being, leading to feelings of loneliness and anxiety. However, Teresa retains her functional independence, enjoys good physical health, and is able to manage her daily needs without assistance. Although her situation presents some elements associated with social frailty, it does not exhibit all the essential attributes of the concept; therefore, it is considered a borderline case.

**Contrary case**

Isabel, 68, lives with her daughter and son-in-law, who are deeply involved in her daily life, sharing household responsibilities and providing her with continuous emotional support. During her working life, Isabel was employed as a salesperson in a small family-owned business and was consistently an active member of her community. She participated in neighborhood activities, collaborated in organizing committees, and supported her children's school activities. She also cultivated a wide network of female neighbors with whom she engaged in recreational and charitable activities. At present, Isabel has managed to maintain that socially active lifestyle. She regularly attends yoga classes, participates in a local reading group, and is a member of a neighborhood volunteer committee. Her support network consists of family members, friends, and neighbors with whom she maintains frequent contact, both in person and through digital media, allowing her to remain connected and feel valued.

Thanks to her constant involvement and the emotional and practical support she receives, Isabel enjoys excellent physical health, emotional stability, and high levels of well-being. Her situation represents the opposite of social frailty, as it displays none of the attributes of the concept: there is no loss of social resources, no isolation, and no difficulties in meeting their basic social needs. According to Walker and Avant, a contrary case is one that does not contain any attributes of the concept under analysis^21^.

The construction and analysis of cases allow for a deeper understanding of the concept of social frailty and its boundaries. The model case clearly illustrates the presence of all essential attributes: loss of networks, diminished participation, difficulties in meeting social needs, and emotional consequences, allowing a visualization of how the concept manifests in practice.The related case presents an intermediate situation, in which some attributes of the concept are present, but protective factors, such as effective family support, prevent the full configuration of social frailty. This case is useful for distinguishing established social frailty from conditions of risk or early social decline, and for recognizing that the phenomenon can manifest gradually rather than through abrupt or total loss. The borderline case highlights the difficulty of classifying certain situations, where signs of isolation or reduced participation are present but without functional loss or complete social disconnection. Such cases challenge the boundaries of the concept and contribute to refining its theoretical definition. Finally, the contrary case confirms the absence of social frailty, offering a clear contrast between the central attributes of the concept and a situation in which resources, bonds, and active participation are preserved. Taken together, these cases reinforce the validity of the analysis and support a more accurate, differentiated, and applicable understanding of the concept in community-dwelling older adults.

**Identifying antecedents and consequences**

**Antecedents**

The literature identifies several factors that precede the onset of social frailty in community-dwelling older adults. Advanced age is one antecedent, as it increases the risk of isolation and dependency^63^. Depressive symptoms^80^ affect motivation and social capacity for social engagement, and cognitive impairment limits active participation in the social contexts^46^.

Declining participation in social and community activities, such as volunteering or visiting family members, is a key factor for social frailty. Physical inactivity and motor dysfunction also contribute to isolation^81^. In addition, the use of substances, such as alcohol and tobacco, affects physical health and the maintenance of social support networks^82^. Recent life events, such as the illness or injury of a spouse, are associated with the development of social frailty as they often trigger isolation^32^. These factors interact in complex ways, increasing the risk of social frailty in older adults.

Evidence suggests that there are significant differences between men and women in the occurrence of social frailty. Several studies show that women tend to score higher on measures of social frailty and are more likely to be classified as socially prefrail or frail^16^. This increased vulnerability may be explained by greater longevity and gender-related differences in health, which appear to increase the risk of frailty among women^28^. Likewise, studies have consistently reported a higher prevalence of women in social frailty indicators^43^, which is consistent with findings that point to a sustained increase in the number of women in the frailest groups^44^.

**Consequences**

Social frailty in older adults has significant consequences for physical, mental, and functional health. It has been associated with an increased risk of depressive symptoms, functional disability, cognitive impairment, and higher mortality according to various studies. Social frailty is associated with the development of depressive symptoms in older adults, affecting their emotional well-being and quality of life^44^,^83^. Additionally, it increases the likelihood of physical disability, which limits autonomy and the ability to perform daily activities^15^,^16^,^84^. Another significant effect of social frailty is cognitive decline, which can lead to dementia or diseases such as Alzheimer's, thereby exacerbating older adults’ dependency^36^,^85^.

Finally, social frailty in older adults is a major risk factor for mortality, as physical frailty and disability increase vulnerability to severe illness, hospitalizations, and health complications^82^,^86^. The interaction among these factors increases the risk of premature death, highlighting the importance of addressing social frailty as a key determinant of health in older adulthood.

**Figure 2:**
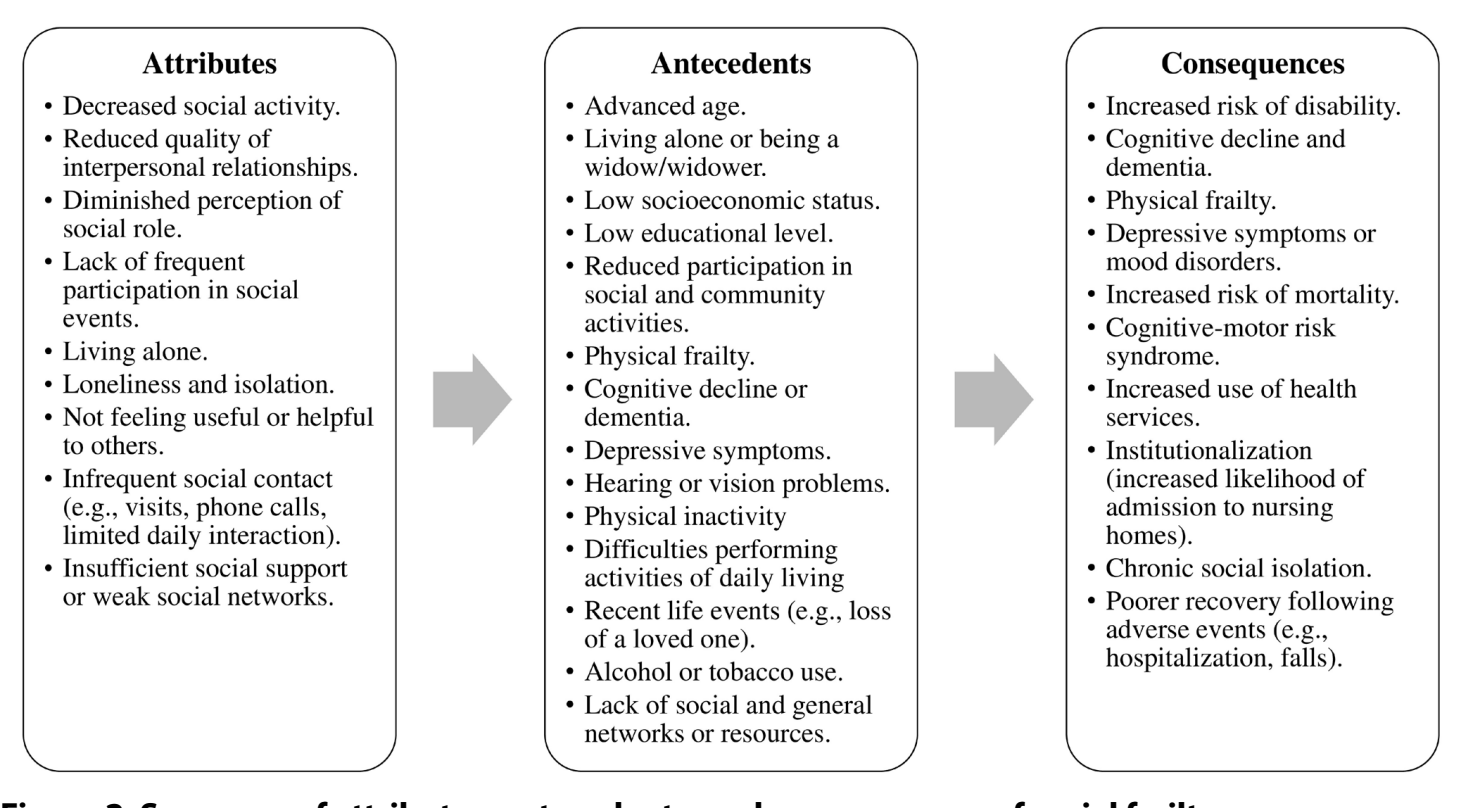
Summary of attributes, antecedents, and consequences of social frailty

*Note: Physical frailty is represented both as an antecedent and as a consequence of social frailty. Empirical evidence identifies it as a prior risk factor (Ma et al., 2018^32^; Li et al., 2024^35^) and also as a subsequent result of the deterioration of social resources and social ties (Makizako et al., 2018^17^; Hamid et al., 2024^27^).*

**Empirical referents**

Although multiple scales have been developed to measure social frailty, many of them have conceptual gaps that limit their usefulness for an in-depth analysis of the phenomenon. In particular, some instruments lack a robust operational definition or fail to explicitly articulate the theoretical foundations underlying their items, which hinders their applicability across different social and cultural contexts. This conceptual gap justifies the need to advance in a concept analysis that strengthens the theoretical and operational bases for future measurements.

The most widely used instrument for assessing social frailty is the scale developed by Makizako et al.^15^, which has been applied in several studies^43^,^46^,^80^,^81^,^86^-^91^. Other methods include the HALFE scale^40^,^92^ scale and the SFS-8 scale^93^,^94^. However, the diversity of approaches and the lack of consensus regarding evaluation criteria highlight the urgent need to develop more comprehensive and culturally adaptable tools that can more accurately capture the complexity and multidimensional nature of social frailty.

## Discussion

Social frailty has been conceptualized as a multidimensional phenomenon reflecting the vulnerability of individuals, especially in old age, to the loss of essential social resources. According to Bunt et al.^14^, it is understood as a continuum of risk in which individuals experience the loss of resources and abilities fundamental to meeting their social needs. This definition is key to preventive medicine and nursing, as it enables the identification of older adults at risk of severe consequences due to inadequate social support.

The present analysis highlights that social frailty is closely linked to factors such as social resources and participation in social activities, which are essential for mitigating the impact of social frailty. According to several studies, social networks and emotional support from family and friends are key in reducing the negative effects of social frailty^95^,^96^. Conversely, lack of social participation and isolation contribute to physical and mental health decline, underscoring the need to promote social integration and active community participation.

The studies reviewed indicate that social frailty is a significant predictor of mortality and cognitive decline in community-dwelling older adults^58^,^82^,^97^. Consistent associations have been identified between social frailty and physical frailty, as well as between lack of support networks and mental health. The absence of adequate social support is associated with increased risk of depression, anxiety, and diminished quality of life^98^.

The concept of social frailty differs from that of social vulnerability. While both concepts share some attributes, social vulnerability addresses a more overall condition of risk. Social vulnerability has a broader scope, whereas social frailty focuses on the loss of social resources and skills essential for well-being in later life^99^. Both concepts share risk factors, such as a lack of social support and low participation; however, social frailty represents a more advanced dysfunction in social ties, directly impacting individual well-being.

The operational definition of social frailty proposed by Makizako et al.^15^, based on indicators like participation in social activities and perceived usefulness in relationships, is widely used in the literature. This approach can serve as a basis for developing accurate measurement tools that assess the degree of social frailty in older adults and facilitate the implementation of appropriate interventions.

This analysis highlights the importance of differentiating between social frailty and social prefrailty. The latter represents an early stage in which social resources remain largely intact, but signs of isolation and vulnerability are already evident. This suggests that early intervention may be critical for preventing progression to more severe forms of social frailty^15^.

The antecedents and consequences of social frailty, such as advanced age, physical limitations, and decreased social participation, provide a framework for understanding the dynamics of social frailty and its implications for public health. Social frailty affects both the physical and mental health of older adults and increases the risk of disability and premature death^100^,^101^. These findings underscore the need for public policies and intervention strategies that promote social integration and community support to mitigate the negative effects of aging.

## Conclusions

The analysis of social frailty in older adults confirms that it is a multidimensional and evolving phenomenon, characterized by the loss or weakening of social resources, reduced participation in meaningful activities, and a decline in the ability to meet basic social needs. This condition, manifested through indicators such as isolation, low perceived social usefulness, and the breakdown of emotional bonds, has direct and measurable consequences on the physical, mental, and functional health of older adults, increasing the risk of disability, cognitive decline, depressive symptoms, and mortality.

Clarifying this still-evolving concept helps to overcome the terminological ambiguity present in some parts of the literature and provides a more coherent theoretical foundation to guide future research, as well as the design of valid and culturally sensitive instruments and specific and effective interventions. Differentiating social frailty from related constructs such as social vulnerability or social prefrailty is essential for establishing tailored courses of action according to specific stages and individual needs.

This study provides a comprehensive, operational, and contextualized definition of social frailty, integrating antecedents, attributes, and consequences from a critical gerontological perspective. The findings reinforce the need for health, well-being, and social protection systems to recognize social frailty as a structural component of comprehensive geriatric assessment. Its explicit inclusion in clinical protocols, practice guidelines, and public policies would foster earlier detection and the development of intersectoral strategies that promote the strengthening of support networks, social participation, and active, inclusive, and dignified aging.
